# Snapshot and crystallographic observations of kinetic and thermodynamic products for NO_2_S_2_ macrocyclic complexes

**DOI:** 10.1107/S2052252517015081

**Published:** 2018-01-01

**Authors:** In-Hyeok Park, Yunji Kang, Eunji Lee, Huiyeong Ju, Seulgi Kim, Sujin Seo, Jong Hwa Jung, Shim Sung Lee

**Affiliations:** a Department of Chemistry and Research Institute of Natural Science, Gyeongsang National University, Jinju 52828, Republic of Korea

**Keywords:** snapshots, kinetic products, thermodynamic products, single-crystal-to-single-crystal transformations, crystal engineering, molecular crystals

## Abstract

Direct observation and structural characterization of a kinetic product and a thermodynamic product for complexes with an NO_2_S_2_ macrocycle (**L**) are reported. **L** reacts with copper(I) iodide to give a mononuclear complex [Cu(**L**)]_2_(Cu_2_I_4_)·2CH_2_Cl_2_ (**1**), featuring three separate units. When cadmium(II) iodide was reacted with **L**, an anion-coordinated complex [Cd(**L**)I]_2_(Cd_2_I_6_)·4CH_3_CN (**2**) with a needle-type crystal shape was formed as the kinetic product. Interestingly, when the needle-type kinetic product was left undisturbed in the mother solution it gradually transformed to the pseudo-dimer complex [Cd_2_(**L**)_2_I_2_](Cd_2_I_6_) (**3**) with a brick-type crystal shape as the thermodynamic product. The dissolution–recrystallization process resulted in the elimination of the lattice solvent molecules (aceto­nitrile) in **2** and the contraction of two neighboring macrocyclic complex units [Cd(**L**)I]^+^, forming the pseudo-dimer **3** *via* an intermolecular Cd⋯I interaction between two monomers. For the entire process from kinetic to thermodynamic products, it was possible to obtain sequential photographic snapshots, single-crystal X-ray structures and powder X-ray diffraction patterns. For the copper(I) and cadmium(II) complexes, competitive NMR results agree with the solid-state data that show copper(I) has a higher affinity for **L** than does cadmium(II).

## Abstract 2

The complexation of an NO_2_S_2_ macrocycle with CdI_2_ offers an opportunity to identify the kinetic and thermodynamic products *via* visual methods because the direct observation and structural characterization of each product were available from sequential snapshots, single-crystal X-ray structures and powder X-ray diffraction patterns.

## 1. Introduction   

**Figure 1:**
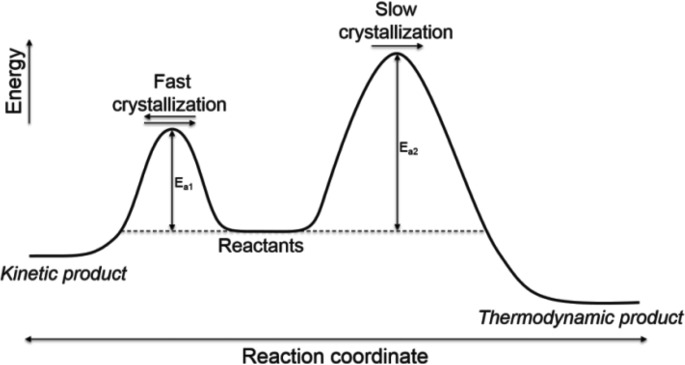
Reaction routes for a kinetic product *via* fast crystallization and a thermodynamic product *via* slow crystallization in the assembly reaction.

Similar to organic reactions, self-assembly of synthetic co­ordination processes affords not only thermodynamic products but also kinetic products when the energy for the latter is trapped in local minima (Percec *et al.*, 2011 ▸; Gammon *et al.*, 2010 ▸; Hwang *et al.*, 2004 ▸; Hasenknopf *et al.*, 1998 ▸). In principle, the reason for the two products is the difference in their activation energy: kinetic products form rapidly and they usually occur at lower temperature, while thermodynamic products form slowly or at higher temperatures (Fig. 1 ▸) (Martí-Rujas & Kawano, 2013 ▸; Martí-Rujas *et al.*, 2011 ▸). The kinetic states in the self-assembly of coordination products play a crucial role in understanding the mechanism and final products as well as the fundamental aspects of functionalization (Percec *et al.*, 2011 ▸; Gammon *et al.*, 2010 ▸; Hwang *et al.*, 2004 ▸; Hasenknopf *et al.*, 1998 ▸; Martí-Rujas & Kawano, 2013 ▸; Martí-Rujas *et al.*, 2011 ▸). However, it is hard to recognize or separate these two products completely and structurally characterize them in the single-crystal state as pure forms due to the difficulty of growing single crystals, because fast precipitation so often leads to the kinetic product. Kawano and co-workers proposed an *ab initio* powder X-ray diffraction (PXRD) approach as an alternative methodology to overcome these difficulties (Martí-Rujas & Kawano, 2013 ▸; Martí-Rujas *et al.*, 2011 ▸). Recently, Ohtsu & Kawano (2017 ▸) highlighted the kinetic effect in self-assembly of coordination networks.

In our previous work, removal of the coordinated or noncoordinated solvent molecules of supramolecular complexes has played a key role in the reaction process between the kinetic and thermodynamic products (Lee *et al.*, 2010 ▸, 2013 ▸; Ju *et al.*, 2015 ▸, 2017 ▸). We recently isolated a 19-membered NO_2_S_2_ macrocycle **L** (see scheme[Chem scheme1]) and a 38-membered macrocycle from the mixed products of the [1:1] and [2:2] cyclization reactions, respectively (Kang *et al.*, 2016 ▸). In complexes with copper(I) iodide, the smaller macrocycle **L** forms a typical mononuclear complex in which all donors in the ring cavity cooperatively bind to the central copper(I) ion (Kang *et al.*, 2016 ▸), while the larger macrocycle affords a tetranuclear bis(macrocycle) complex adopting a double-decker structure as a first example of this type (Kang *et al.*, 2016 ▸). Sulfur donors in crown-type macrocycles have a tendency to lead metal coordination outside the cavity (*exo*-coordination) to form discrete or infinite complexes with some thia­philic metal ions (Lee, Kim *et al.*, 2008 ▸; Lee, Lee *et al.*, 2008 ▸; Park *et al.*, 2012 ▸, 2014 ▸). However, the presence of a pyridine subunit in **L** is expected to locate the metal ion inside the cavity (Lee *et al.*, 2016 ▸, 2015 ▸; Drahoš *et al.*, 2017 ▸; Fedorov *et al.*, 2017 ▸). Considering the binding affinity of **L** to metal ions with respect to their donor basicity, we have employed copper(I) and cadmium(II) ions (iodide form).
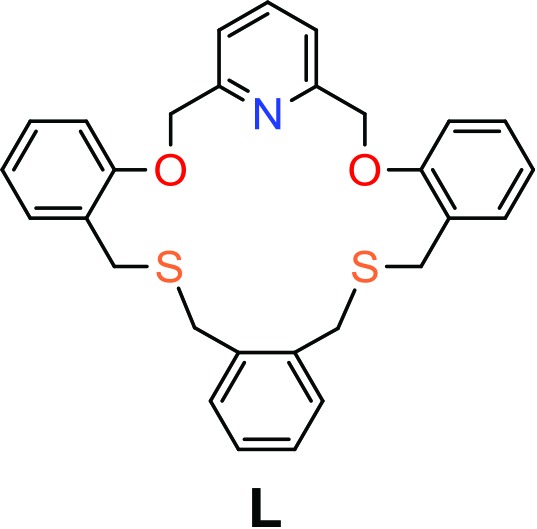


In the present work, the ligand **L** yields two products of CdI_2_ with different crystal habits depending on the reaction time. Sometimes a mixture of crystalline products can be separated manually in pure form under a microscope because of their different crystal habits (Moreno-Calvo *et al.*, 2010 ▸; Ryu *et al.*, 2014 ▸; Park *et al.*, 2014 ▸). In view of these intriguing results we decided to follow the reaction process more closely, in order to obtain insight into the structural and mechanical factors that may act. Fortunately, we were able to obtain a series of snapshot images for the two products with different crystal habits exhibiting growth–dissolution–recrystallization depending on the time between the kinetic and thermodynamic control processes. Here, we report several complexes of **L** which offer an opportunity to identify the kinetic and thermodynamic products *via* visual methods because of the slow reaction process and different crystal habits. The details are discussed below.

## 2. Results and discussion   

### 2.1. Copper(I) iodide complex **1**   

**Figure 2:**
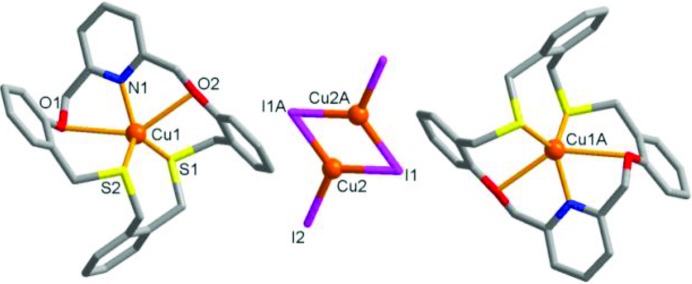
The molecular structure of **1**, [Cu(**L**)]_2_(Cu_2_I_4_)·2CH_2_Cl_2_, showing the three separate metal-containing units. The noncoordinated solvent molecule has been omitted.

**L** was prepared as described by us previously (Kang *et al.*, 2016 ▸). The reaction of **L** in di­chloro­methane with CuI in aceto­nitrile afforded a colorless crystalline product, **1**. The X-ray analysis revealed that **1** features three separate metal-containing units with the formula [Cu(**L**)]_2_(Cu_2_I_4_)·2CH_2_Cl_2_: two macrocyclic copper(I) complex cations [Cu(**L**)]^+^ and one anionic copper(I) iodide cluster (Cu_2_I_4_)^2−^ (Fig. 2 ▸ and Table 1 ▸). Since the inversion center is located in the middle of the anionic cluster, the asymmetric unit contains one macrocyclic cation and one half of the cluster. In the macrocyclic complex cation, the copper(I) center binds to all donors of **L** in a twisted conformation, adopting a distorted square-pyramidal coordination geometry (*τ* = 0.25; Addison *et al.*, 1984 ▸) with atoms N1, O1, O2 and S1 defining a square plane, and atom S2 the apex. The Cu_2_I_4_ cluster is planar, similar to other examples reported (Haase *et al.*, 2011 ▸; Basu *et al.*, 1987 ▸; Kia *et al.*, 2007 ▸; Bhaduri *et al.*, 1991 ▸). The Cu1—N1 bond distance [2.032 (8) Å] is consistent with the strong coordination of copper(I) toward the pyridine N atom. The Cu—S bond distances [Cu1—S1 = 2.274 (3) Å and Cu1—S2 = 2.255 (3) Å] are typical and the Cu—O bond distances [Cu1—O1 = 2.712 (7) Å and Cu1—O2 = 2.657 (8) Å] are also normal.

Recently, we have reported a copper(I) iodide complex of **L** with the formula [Cu(**L**)]I_3_·ether isolated from di­chloro­methane/aceto­nitrile/ether (Kang *et al.*, 2016 ▸). In this case, the cationic complex part [Cu(**L**)]^+^ is also five-coordinate, adopting a distorted square-pyramidal geometry (*τ* = 0.31), but one triiodide ion (I_3_^−^) exists as a counteranion due to the partial oxidation of copper(I) to copper(II) (Kang *et al.*, 2016 ▸; Lee *et al.*, 2017 ▸).

### 2.2. Cadmium(II) iodide complexes: kinetic product **2** and thermodynamic product **3**   

**Figure 3:**
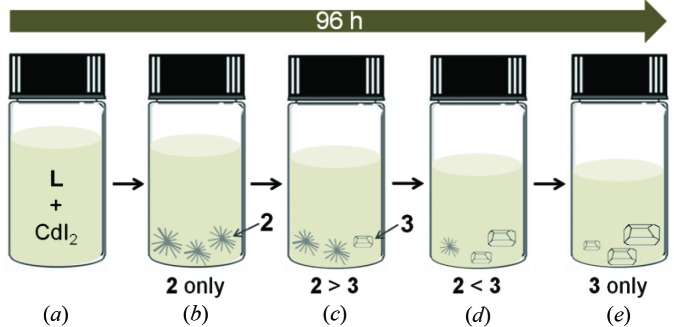
Cartoon presentation of time-dependent crystal growth, dissolution and recrystallization in the mother solution: **2** forms needles and **3** forms bricks.

It is notable that the complexation of **L** with CdI_2_ yields two products whose crystal shapes are different, **2** forming needles and **3** forming bricks, and their compositions change depending on the reaction time (Fig. 3 ▸). In these observations, we provide decisive evidence for the stepwise formation of a kinetic product and a thermodynamic product, not only to the naked eye but also *via* a crystallographic approach. The details are discussed in the next section.

**Figure 4:**
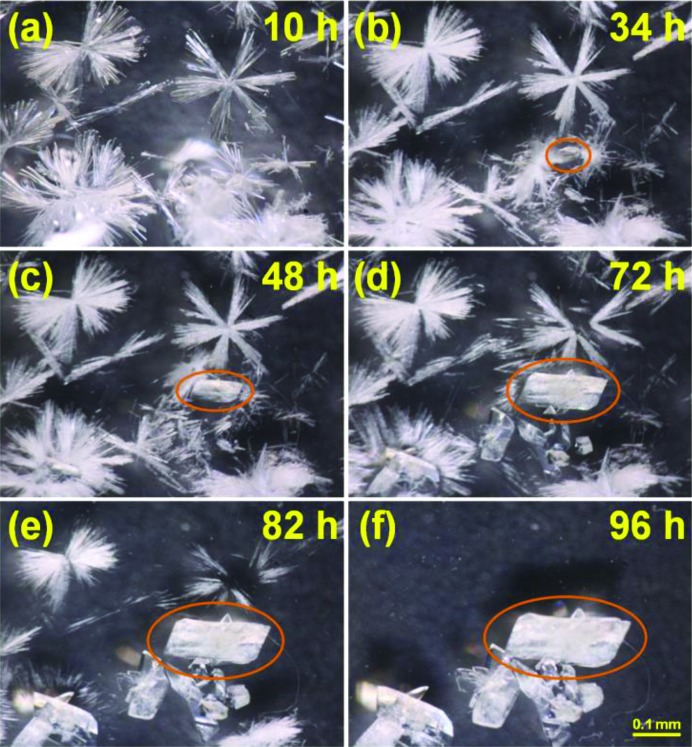
Time-series snapshot optical microscope images for **2** and **3** in the mother solution. (*a*) Needle-shaped crystals of **2** obtained after 10 h. (*b*) Brick-shaped crystals of **3** appear (orange circles). (*c*)–(*f*) The needles of **2** disappear gradually and the number and size of the bricks of **3** increase. (*g*) Only the brick-shaped crystals of **3** exist after 96 h.

When **L** was reacted with CdI_2_ in di­chloro­methane/aceto­nitrile, as mentioned above, two products, **2** and **3**, identified by eye due to their different crystal habits, were isolated. Thus, time-series snapshots for the single-crystal growth process were recorded by optical microscope photography (Fig. 4 ▸, see also Fig. S1 and Movie S1 in the supporting information). Indeed, slow evaporation of this reaction mixture for 10 h gave a colorless needle-shaped crystalline product **2** (Fig. 4 ▸ *a*). When the needle-shaped product **2** was left undisturbed in the mother solution, a small brick-shaped crystalline product **3** appeared after 34 h (Fig. 4 ▸ *b*). Thereafter, the size and number of crystals of the brick-shaped product **3** increased and the needle-shaped crystals of **2** gradually disappeared (Figs. 4 ▸ *c*–4 ▸ *e*). After 96 h, **2** disappeared completely and only **3** was present (Fig. 4 ▸ *f*).

**Figure 5:**
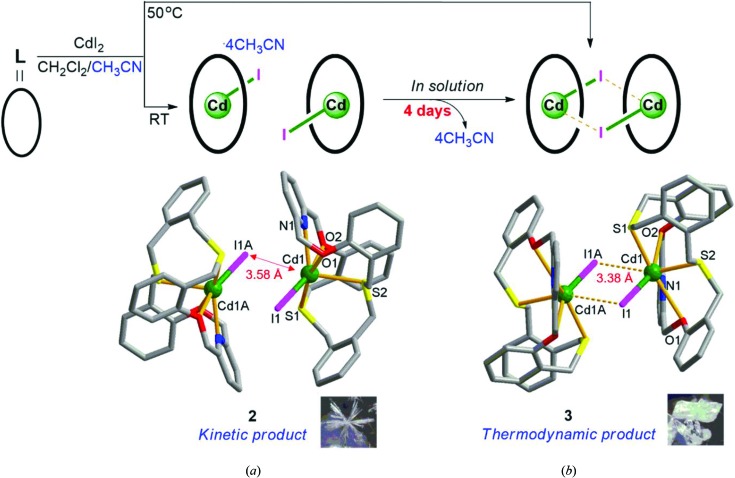
Molecular structures of (*a*) **2**, [Cd(**L**)I]_2_(Cd_2_I_6_)·4CH_3_CN, and (*b*) **3**, [Cd_2_(**L**)_2_I_2_](Cd_2_I_6_), isolated from the dissolution–recrystallization process involving the loss of lattice solvent molecules from **2** (aceto­nitrile, not shown) in solution. The anionic (Cd_2_I_6_)^2−^ clusters in both products have been omitted. The distances between Cd1 and I1*A* are 3.5768(8) Å (red arrow) in **2** and 3.3827(8) Å (dashed lines) in **3**.

**Figure 6:**
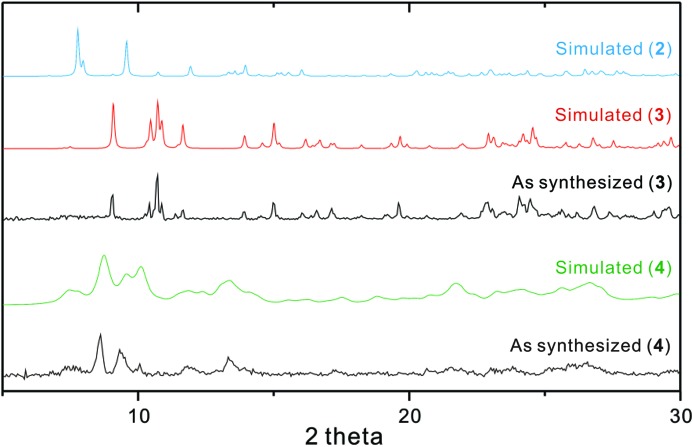
The PXRD patterns of **2**, **3** and **4**.

Single-crystal X-ray analysis revealed that the two observed types of crystals, **2** and **3**, have different cell parameters, compositions and structures (Moreno-Calvo *et al.*, 2010 ▸; Ryu *et al.*, 2014 ▸; Park *et al.*, 2014 ▸). For instance, the needle-shaped product, **2**, crystallizes in the monoclinic space group *C*2/*c* with the formula [Cd(**L**)I]_2_(Cd_2_I_6_)·4CH_3_CN (Fig. 5 ▸ *a*, and Table S2 in the supporting information), while the brick-shaped product, **3**, crystallizes in the triclinic space group 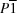 with the formula [Cd_2_(**L**)_2_I_2_](Cd_2_I_6_) (Fig. 5 ▸ *b*, and Table S3 in the supporting information]. Comparison of the PXRD patterns for each separate product and the simulated patterns for the corresponding single crystals indicates that the two species are effectively separate, showing bulk purity (Fig. 6 ▸). Even though both complexes share some common features, it is of great interest to compare these two structures to reveal what happens during the reaction process in solution.

Again, complex **2** contains three separate metal-containing units: two macrocyclic complex cation units [Cd(**L**)I]^+^ and one anionic cadmium(II) iodide cluster unit (Cd_2_I_6_)^2−^ (not shown in Fig. 5 ▸ *a*). In **2**, the two macrocyclic complex units face each other with a Cd1⋯I1*A* distance of 3.5768 (8) Å, which is similar to the sum of the van der Waals radii *r*_VDW_ (3.56 Å; Bondi, 1964 ▸) (Fig. 5 ▸ *a*). The Cd^II^ center in the macrocyclic complex unit is six-coordinate, being bound to all five donors from **L** which adopts a bent and twisted conformation. The coordination sphere is completed by one iodide anion, with the Cd1—I1 bond distance being 2.7422 (7) Å.

**Figure 7:**
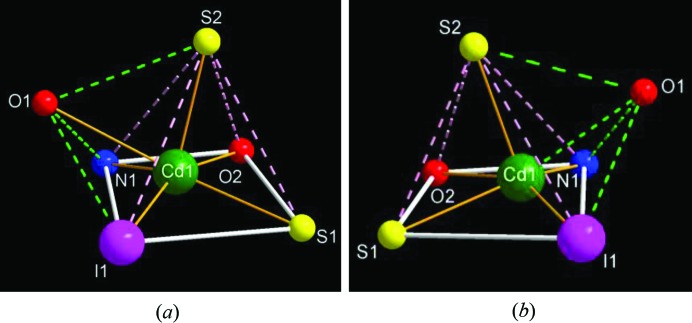
The coordination geometry of the cadmium(II) center in (*a*) **2** and (*b*) **3**, showing a distorted monocapped square-pyramidal arrangement.

The key structural feature of **3** is its pseudo-dimeric form *via* the associated change in the Cd1*A*⋯I1 distance from 3.5768 (8) to 3.3827 (8) Å; the latter value is shorter than its *r*_VDW_ (3.56 Å) (Fig. 5 ▸ *b*). Large conformation changes in the macrocycle are also observed due to the removal of the lattice solvent molecules and the dimeric interaction. The metal centers in both **2** and **3** are six-coordinate, adopting a monocapped square-pyramidal geometry (Fig. 7 ▸). However, the rearrangement from **2** to **3** involves geometric changes to the coordination sphere as well as conformational changes of **L**. Considering the interatomic distances between Cd1*A* and I1, it might be concluded that the removal of the lattice aceto­nitrile molecules in **2** induces the contraction of the molecule, resulting in the pseudo-dimer formation of **3**.

Some modified crystallization approaches were also carried out, affording isolation of brick-shaped **3** directly from the reaction mixture (Lee *et al.*, 2010 ▸, 2013 ▸; Ju *et al.*, 2015 ▸, 2017 ▸). For example, under identical reaction conditions but with stirring at 50°C for 20 min (Fig. 5 ▸), a solid precipitate was obtained. The PXRD pattern confirmed that this solid is pure complex **3** (Fig. 6 ▸), suggesting direct preparation at the higher temperature as depicted in Fig. 1 ▸. Alternatively, when several single crystals of **3** were added as seeds to the corresponding freshly prepared reaction mixture solution, a large quantity of brick-shaped **3** was obtained *via* extra crystal nucleation and crystal growth without the appearance of the metastable species **2**. Furthermore, solvent diffusion of a di­chloro­methane solution of **L** into an aceto­nitrile solution of cadmium(II) iodide gave only product **3**.

When we consider the snapshot images together with the high temperature and the seeding approaches showing direct preparation of **3**, the observed crystal habit changing from **2** to **3** in the mother solution can be understood in terms of the reaction process, from kinetic product **2** to thermodynamic product **3**. Earlier, our time-dependent crystallization experiments showed that a one-dimensional silver(I) perchlorate coordination polymer of an O_2_S_2_ macrocycle is a kinetic product and transforms into a thermodynamically more stable two-dimensional network (Lee *et al.*, 2010 ▸). More recently, our group introduced a disilver(I) solvato-complex of a 40-membered N_4_O_4_S_4_ macrocycle as a kinetic product which shows rearrangement to the desolvated thermodynamic product (Lee *et al.*, 2013 ▸).

### 2.3. A single-crystal-to-single-crystal transformation of **2** in air   

**Figure 8:**
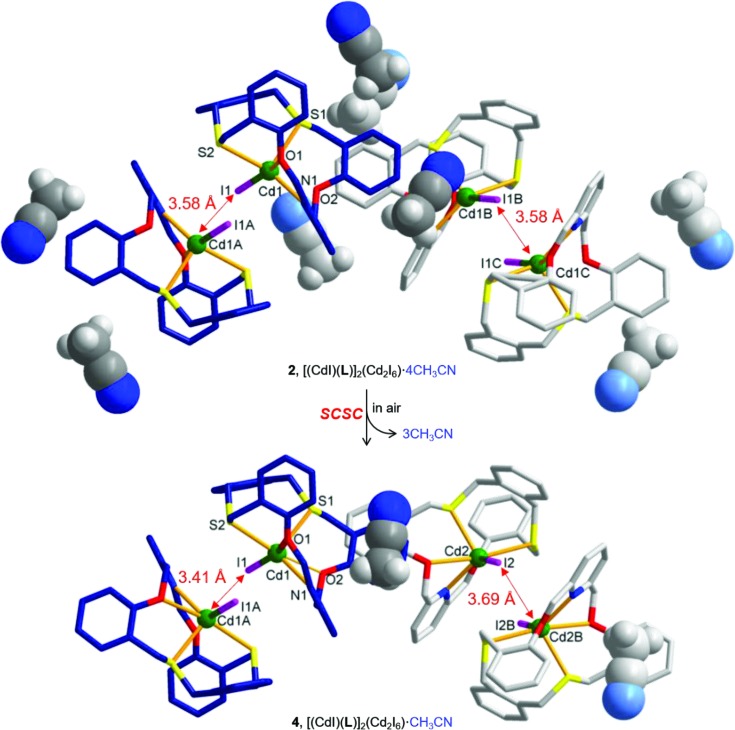
The SCSC transformation of **2** (top) to **4** (bottom) *via* the partial removal of the lattice solvent molecules. The anionic clusters (Cd_2_I_6_)^2−^ have been omitted.

In air, unlike in the mother solution, kinetic product **2**, [Cd(**L**)I]_2_(Cd_2_I_6_)·4CH_3_CN, showed a different transformation behavior (Fig. 8 ▸). In **2**, as mentioned, four lattice aceto­nitrile molecules are trapped in each formula unit. When colorless crystals of **2** were filtered off and isolated from the mother solution and kept in air, we confirmed that three lattice aceto­nitrile molecules were removed by sublimation within several hours to give compound **4**, [Cd(**L**)I]_2_[(Cd_2_I_6_)·CH_3_CN] (Fig. 8 ▸). During this partial sublimation process, the crystals lost transparency but it was possible to obtain the single-crystal structure. Notably, the removal of the lattice solvent molecules by partial sublimation also induced some structural changes in the complex.

Unlike the parent complex **2**, for example, complex **4** has two crystallographically different Cd^II^ atoms (Cd1 and Cd2). In addition, some changes in the pairwise interaction between two macrocyclic complex units were observed: the distance between Cd1*A* and I1 in **2** is 3.5768 (8) Å, while it is 3.415 (7) Å (Cd1*A*⋯I1) and 3.690 (7) Å (Cd2⋯I2*B*) in **4**. Due to the sublimation of the solvent molecules followed by the structural change, **4** shows a high *R*_1_ value (0.1848; Table 1 ▸). Recently, we reported some examples of the sliding re­arrangement of a double-decker type complex (Kang *et al.*, 2016 ▸) of the [2:2] cyclization analogue of **L** and a one-dimensional coordination polymer of the bis-di­thia­macrocycle (Kim *et al.*, 2016 ▸) *via* a single-crystal-to-single-crystal (SCSC) transformation upon desolvation in air. The homogeneity of **4** was confirmed by PXRD patterns (Fig. 6 ▸). Exposure of **4** to aceto­nitrile liquid and vapor results in no structural change, suggesting that the above structural transformation is not reversible.

### 2.4. Complexation with a mixture of CuI and CdI_2_   

**Figure 9:**
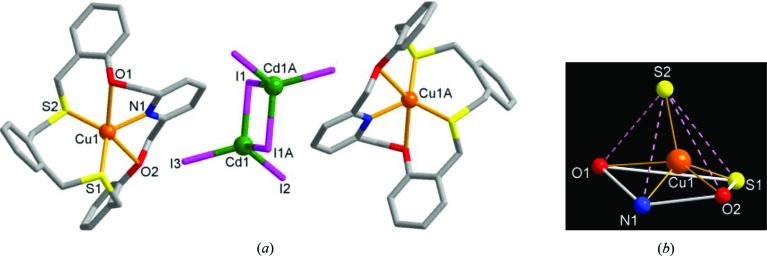
The molecular structure of **5**, [Cu(**L**)]_2_(Cd_2_I_6_), showing the three separate parts. (*a*) A general view and (*b*) the distorted square-pyramidal coordination geometry of the copper(I) center. [Symmetry code: (*A*) 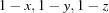.]

As an extension of the above homonuclear copper(I) iodide complex **1** and cadmium(II) iodide complexes **2**–**4**, we investigated the relative reactivity of copper(I) and cadmium(II) as soft acids towards **L** in both the solid and solution states. When a mixture of copper(I) iodide and cadmium(II) iodide was reacted with **L** in aceto­nitrile/di­chloro­methane, a yellow block-shaped crystalline product **5** was isolated. X-ray analysis revealed that **5** contains three separate metal-containing parts in the formula [Cu(**L**)]_2_(Cd_2_I_6_) (Fig. 9 ▸ *a*): two macrocyclic monocopper(I) complex cations and one (Cd_2_I_6_)^2−^ cluster anion, indicating a preferential coordination affinity of copper(I) over cadmium(II) towards **L**. Since the inversion center exists in the middle of the (Cd_2_I_6_)^2−^ cluster, the asymmetric unit contains one macrocyclic copper(I) complex unit and half a cluster. The copper(I) center inside the cavity is five-coordinate, being bound by all the donors from **L** in a twisted conformation. The Cu^I^ coordination geometry is a distorted square-pyramidal geometry (τ = 0.29) with donors S1, O1, O2 and N1 of **L** defining a distorted square plane and the S2 donor in an axial position (Fig. 9 ▸ *b*).

### 2.5. Comparative NMR study of Cu^I^ and Cd^II^ complexation   

**Figure 10:**
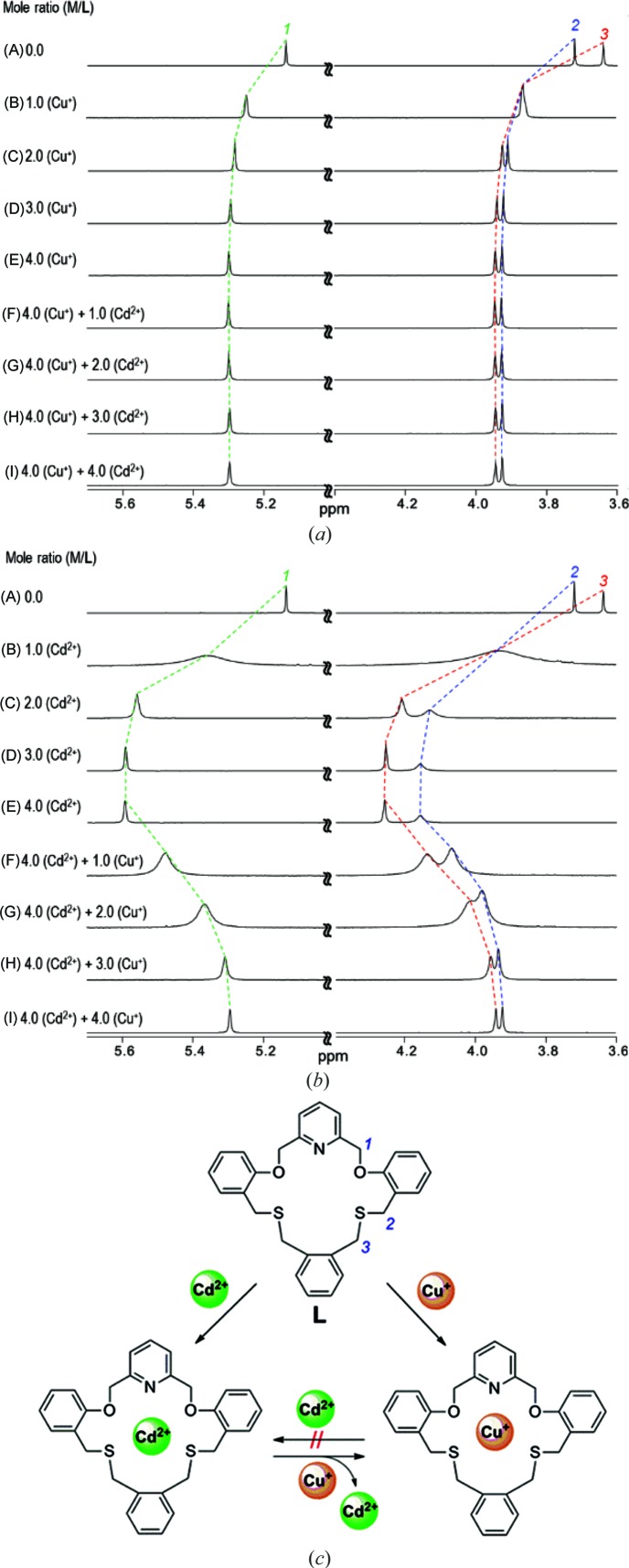
^1^H NMR spectra of the aliphatic region for **L** in CD_3_CN *via* stepwise addition of (*a*) Cd^2+^ and Cu^+^, and (*b*) Cu^+^ and Cd^2+^. [**L**] = 5 m*M*. (*c*) The proposed complexation equilibria between the corresponding species in solution.

Comparative NMR experiments for the competition reactions of copper(I) and cadmium(II) toward **L** were also performed. In Fig. 10 ▸, the signals of the aliphatic protons (H_1_–H_3_) in **L** are well resolved and identified. As shown in Fig. 10 ▸(*a*) line (B), the addition of 1–4 equivalents of copper(I) causes downfield shifts for H_1_–H_3_ of 0.15–0.3 p.p.m. and indicates that complexation with fast ligand exchange is occurring on the NMR time scale. In this case, the chemical shift changes are H_3_ > H_2_ > H_1_, indicating that copper(I) favors binding to the S donors rather than to the O donors. Further addition of cadmium(II) (1–4 equivalents) led to no significant chemical shift changes [Fig. 10 ▸ *a* lines (F) and (I)], suggesting that the copper(I) complex formed earlier is maintained and no further reaction occurs (as proposed in Fig. 10 ▸ *c*).

Comparative NMR experiments for the above competition reaction were also performed in the reverse order of salt addition [that is, cadmium(II) followed by copper(I)]. As shown in Fig. 10 ▸(*b*) lines (A) and (E), the cadmium(II) complexation proceeds by two steps. First, the addition of one equivalent of cadmium(II) causes downfield shifts for H_1_–H_3_. On addition of another one equivalent of cadmium(II), further downfield shifts of each peak were observed, in keeping with the formation of a dicadmium(II) species [Cd_2_ **L**]^4+^ (Fig. 10 ▸ *b*). On addition of one equivalent of copper(I), larger upfield shifts occur and the spectral pattern in Fig. 10 ▸(*a*) line (I) becomes the same as that shown in Fig. 10 ▸(*b*) line (I), suggesting that the respective reactions finally reach the formation of the monocopper(I) species [Cu**L**]^+^ (Fig. 10 ▸ *c*, anticlockwise direction starting from **L**). Again, this result agrees with the solid-state data in showing that copper(I) has a higher affinity for **L** than does cadmium(II). Considering the size effect of the metal ions [Cd^II^ (4*d* ^10^) is slightly larger than Cu^I^ (3*d* ^10^)] on their affinity towards the 19-membered ring cavity of **L**, Cd^II^ is expected to have a higher affinity, unlike the results obtained from the X-ray and NMR data in this work. Consequently, the greater thia­philic nature of Cu^I^ than of Cd^II^ could be the main reason associated with the shorter bond distances of Cu^I^—S (2.25–2.27 Å) in **1** than those of Cd^II^—S (2.67–2.74 Å) in **2**.

## 3. Conclusions   

In summary, sequential photographic snapshots, single-crystal X-ray structures and PXRD patterns of cadmium(II) iodide complexes of an NO_2_S_3_ macrocycle enable us to identify kinetic and thermodynamic products. From the visual data, it was found that the reaction process from the kinetic product to the thermodynamic product resulted in the elimination of the lattice solvents and the contraction of two facing macrocyclic complex units, resulting in the formation of a pseudo-dimer *via* a Cd⋯I interaction. In the competition reactions, **L** shows preferential complexation behavior towards copper(I) over cadmium(II) in both solid and solution states.

## 4. Experimental   

### 4.1. General   

All chemicals were purchased from commercial sources and used as received. All solvents used were of reagent grade. Elemental analyses were carried out on a LECO CHNS-932 elemental analyzer. Thermogravimetric analyses were recorded on a TA Instruments TGA-Q50 thermogravimetric analyzer. The FT–IR spectra were recorded using a Thermo Fisher Scientific Nicolet *i*S 10 FT–IR spectrometer with KBr pellets.

### 4.2. Preparation of [Cu(L)]_2_(Cu_2_I_4_)·2CH_2_Cl_2_, **1**   

CuI (12 mg, 0.062 mmol) was dissolved in methanol (l.0 ml) and added to a solution of **L** (10 mg, 0.021 mmol) in di­chloro­methane (l.0 ml). A white precipitate formed immediately and this was filtered off. Colorless crystalline **1** suitable for X-ray analysis was obtained by vapor diffusion of di­ethyl ether into a dimethylformamide (0.5 ml) solution of the precipitate (yield 35%). Analysis, calculated for C_60_H_58_Cl_4_Cu_4_I_4_N_2_O_4_S_4_: C 38.98, H 3.10, N 1.54, S 7.05%; found: C 39.23, H 3.27, N 1.44, S 6.83%. IR (KBr pellet, ν, cm^−1^): 2925, 2855, 1719, 1655, 1560, 1459, 1377, 1341, 1296, 1245, 1219, 1186, 1159, 1106, 1048, 1027, 807, 753.

### 4.3. Preparation of [Cd(L)I]_2_(Cd_2_I_6_)·4CH_3_CN, **2**, and [Cd_2_(L)_2_I_2_](Cd_2_I_6_), **3**   

A solution of CdI_2_ (24 mg, 0.066 mmol) in aceto­nitrile (0.5 ml) was added to a solution of **L** (10 mg, 0.021 mmol) in di­chloro­methane (0.5 ml). Slow evaporation of the solution afforded two kinds of crystals: at the beginning (within 2 d) colorless needle-shaped crystals of **2** formed in the vial, which converted to the pale-yellow block-shaped crystals of **3** after 4 d. Separately, white precipitates of **2** and **3** were obtained from a reaction mixture of **L** and CdI_2_ in aceto­nitrile/di­chloro­methane at room temperature and 50°C for 10 min, respectively. For **3**, yield 70%. Analysis, calculated for C_58_H_54_Cd_4_I_8_N_2_O_4_S_4_: C 28.59, H 2.23, N 1.15, S 5.26%; found: C 28.47, H 2.14, N 1.35, S 5.45%. IR (KBr pellet, ν, cm^−1^): 2958, 2920, 2863, 1925, 1719, 1686, 1604, 1579, 1560, 1543, 1508, 1490, 1455, 1440, 1400, 1385, 1372, 1290, 1277, 1246, 1220, 1181, 1161, 1111, 1099, 1047, 1032, 943, 902, 869, 842.

### 4.4. Preparation of [Cd(L)I]_2_(Cd_2_I_6_)_2_·CH_3_CN, **4**   

Single crystals of **4** were obtained from single crystals of **2** which had been kept at room temperature for 10 min in air. Analysis, calculated for C_120_H_114_Cd_8_I_16_N_6_O_8_S_8_: C 29.09, H 2.32, N 1.70, S 5.18%; found: C 28.75, H 2.18, N 1.24, S 5.44%. IR (KBr pellet, ν, cm^−1^): 3051, 2957, 2919, 2344, 1719, 1638, 1604, 1579, 1560, 1543, 1490, 1454, 1440, 1412, 1400, 1385, 1371, 1292, 1276, 1246, 1220, 1193, 1180, 1160, 1111, 1098, 1047, 1032, 1012, 902, 867, 841, 807.

### 4.5. Preparation of [Cu(L)]_2_(Cd_2_I_6_), **5**   

CuI (12 mg, 0.062 mmol) and CdI_2_ (24 mg, 0.066 mmol) were dissolved in aceto­nitrile (l.0 ml) and the solution was layered on a solution of **L** (10 mg, 0.08 mmol) in di­chloro­methane (l.0 ml). The (layered) mixture afforded a yellow crystalline product, **5**, suitable for X-ray analysis (yield 45%). Analysis, calculated for C_58_H_54_Cd_2_Cu_2_I_6_N_2_O_4_S_4_: C 33.42, H 2.61, N 1.34, S 6.15%; found: C 33.77, H 2.53, N 1.28, S 6.41%. IR (KBr pellet, ν, cm^−1^): 2966, 2926, 2855, 1871, 1735, 1655, 1560, 1491, 1459, 1490, 1459, 1253, 1244, 1218, 1188, 1161, 1106, 1075, 1048, 1023, 958, 939, 908, 758, 742.

### 4.6. X-ray crystallographic analysis   

Crystal data for **1**–**5** were collected at 173 K on a Bruker SMART APEXII ULTRA diffractometer equipped with graphite monochromated Mo *K*α radiation (λ = 0.71073 Å) generated by a rotating anode. The cell parameters for the compounds were obtained from a least-squares refinement of the spot (from 36 collected frames). Data collection, data reduction and absorption correction were carried out using the software package *APEX2* (Bruker, 2008 ▸). All calculations for the structure determination were carried out using the *SHELXTL* package (Bruker, 2001 ▸). In all cases, all non-hydrogen atoms were refined anisotropically, and all hydrogen atoms were placed in idealized positions and refined iso­tropically in a riding manner along with their respective parent atoms. Due to the sublimation of the solvent molecules (aceto­nitrile) of **2** followed by a structural change, **4** shows relatively high *R* values. Since the remaining lattice solvent molecules (four aceto­nitrile molecules in **2** and one aceto­nitrile molecule in **4**) are highly disordered, the contribution of solvent electron density was removed using the *SQUEEZE* routine in *PLATON* (Spek, 2009 ▸). Several *SHELXTL* restraints were used in the refinements of **1** and **4**. In **1**, SADI was used to keep within a reasonable geometry for the non-disordered part of the di­chloro­methane molecule. In **4**, SADI, DFIX, SIMU and soft constraints were used to keep within a reasonable geometry for the non-disordered part of the macrocyclic ligand. Relevant crystal data collection and refinement data for the crystal structures of **1**–**5** are summarized in Table 1 ▸, and in Tables S1–S5 in the supporting information.

## Supplementary Material

Crystal structure: contains datablock(s) 1, 2, 3, 4, 5. DOI: 10.1107/S2052252517015081/yc5012sup1.cif

Structure factors: contains datablock(s) 1. DOI: 10.1107/S2052252517015081/yc50121sup2.hkl

Structure factors: contains datablock(s) 2. DOI: 10.1107/S2052252517015081/yc50122sup3.hkl

Structure factors: contains datablock(s) 3. DOI: 10.1107/S2052252517015081/yc50123sup4.hkl

Structure factors: contains datablock(s) 4. DOI: 10.1107/S2052252517015081/yc50124sup5.hkl

Structure factors: contains datablock(s) 5. DOI: 10.1107/S2052252517015081/yc50125sup6.hkl

Time-series snapshots for the single-crystal growth process, Fig. S1. DOI: 10.1107/S2052252517015081/yc5012sup7.gif

Time-series snapshots for the single-crystal growth process, Movie S1. DOI: 10.1107/S2052252517015081/yc5012sup8.mov

Additional tables. DOI: 10.1107/S2052252517015081/yc5012sup9.pdf

CCDC references: 1566938, 1566939, 1566940, 1566941, 1566942

## Figures and Tables

**Table 1:** Crystallographic data and refinement parameters

	**1**	**2**	**3**	**4**	**5**
CCDC refcode	1566938	1566939	1566940	1566941	1566942
Formula	C_30_H_29_Cl_2_Cu_2_I_2_NO_2_ S_2_	C_29_H_27_Cd_2_I_4_NO_2_S_2_	C_58_H_54_Cd_4_I_8_N_2_O_4_S	C_116_H_106_Cd_8_I_16_N_4_O_8_S_8_	C_29_H_27_CdCuI_3_NO_2_S_2_
Formula weight	951.44	1218.03	2436.07	4870.12	1042.28
Crystal system	Triclinic	Monoclinic	Triclinic	Triclinic	Triclinic
Space group	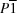	*C*2/*c*	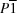	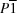	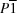
*a* (Å)	11.7925 (4)	23.3017 (6)	11.660 (3)	11.3166 (11)	8.9645 (2)
*b* (Å)	11.9665 (4)	13.4569 (4)	12.147 (3)	13.4404 (15)	14.4098 (3)
*c* (Å)	13.4375 (4)	26.9935 (7)	12.585 (4)	24.586 (2)	14.8826 (3)
α (°)	93.290 (2)	90	75.142 (11)	86.655 (7)	65.8530 (10)
β (°)	95.681 (2)	102.9630 (10)	87.056 (13)	89.396 (6)	73.3860 (10)
γ (°)	118.683 (2)	90	86.996 (13)	78.371 (7)	81.5460 (10)
*V* (Å^3^)	1643.38 (10)	8248.6 (4)	1719.2	3656.6 (6)	1680.04 (6)
*Z*	2	8	1	1	2
*D*_calc_ (Mg m^−3^)	1.923	2.092	2.353	2.212	2.060
μ (mm^−1^)	3.491	4.158	4.977	4.680	4.172
2θ_max_ (°)	52.00	52.00	52.00	48.00	52.00
Reflections collected	22525	36289	28690	23781	28556
Independent reflections	6174 (*R*_int_ = 0.0384)	8103 (*R*_int_ = 0.0499)	6745 (*R*_int_ = 0.0283)	8432 (*R*_int_ = 0.0842)	6602 (*R*_int_ = 0.0254)
Goodness-of-fit on *F* ^2^	1.113	1.112	1.062	1.129	1.042
*R*_1_, *wR*_2_ [*I* > 2σ(*I*)]	0.0738, 0.2191	0.0432, 0.0992	0.0205, 0.0440	0.1848, 0.4191	0.0168, 0.0376
*R*_1_, *wR*_2_ (all data)	0.0935, 0.2336	0.0535, 0.1025	0.0247, 0.0457	0.2147, 0.4304	0.0194, 0.0388
