# Supplementary figures and images for: Snapshot and crystallographic observations of kinetic and thermodynamic products for NO2S2 macrocyclic complexes
Source: IUCrJ. 2018 Jan 1;5(Pt 1):45–53. doi: 10.1107/S2052252517015081 (PMC5755576; doi:10.1107/S2052252517015081)

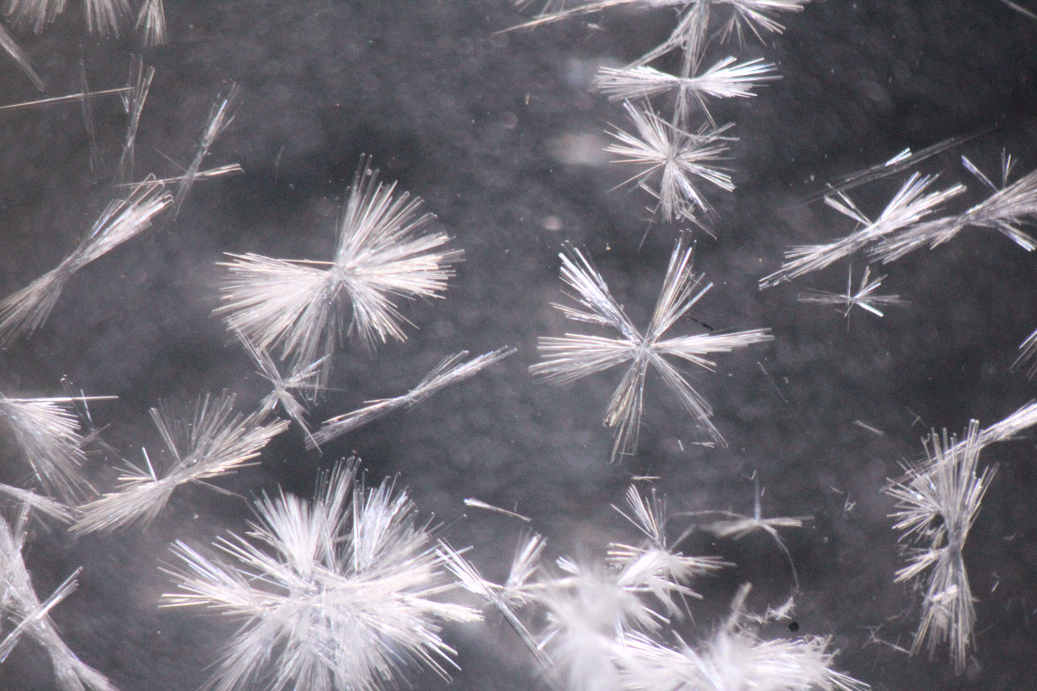

Supplement: Supplementary file 7 [file m-05-00045-sup7.gif]
