# Supplementary material for: Snapshot and crystallographic observations of kinetic and thermodynamic products for NO2S2 macrocyclic complexes
Source: IUCrJ. 2018 Jan 1;5(Pt 1):45–53. doi: 10.1107/S2052252517015081 (PMC5755576; doi:10.1107/S2052252517015081)
Supplement: Supplementary file 9 [file m-05-00045-sup9.pdf]

IUCrJ

Volume 5 (2018)

Supporting information for article:

Snapshot and crystallographic observations of kinetic and thermodynamic products for NO2S2 macrocyclic complexes

In-Hyeok Park, Yunji Kang, Eunji Lee, Huiyeong Ju, Seulgi Kim, Sujin Seo, Jong Hwa Jung and Shim Sung Lee

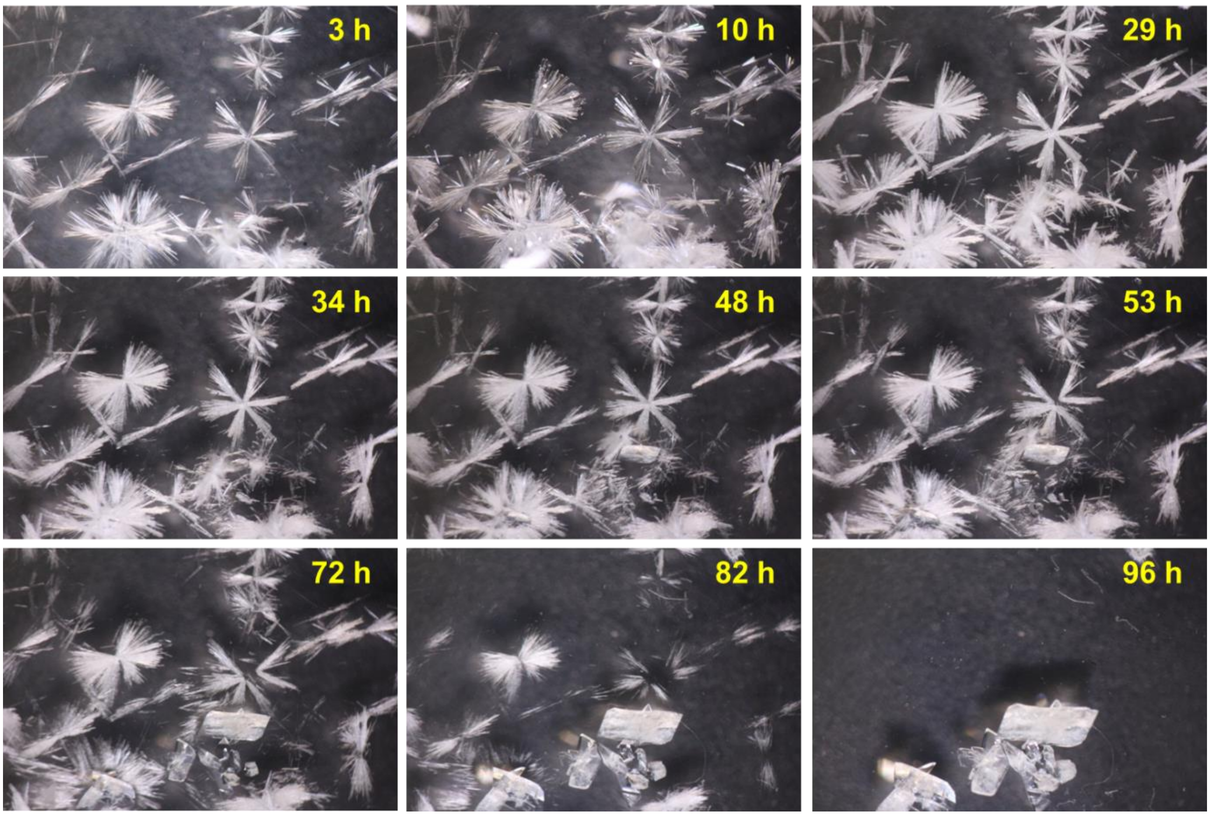

Figure S1 Snapshot photographs from single crystals of **2** (kinetic product) to **3** (thermodynamic product) in reaction mixture at room temperature.

Table S1 Selected bond distances (Å) and bond angles (°) for **1**[a]

Cu1-S1	2.274(3)	Cu1-S2	2.255(3)
Cu1-O1	2.712(7)	Cu1-O2	2.657(8)
Cu1-N1	2.032(8)	Cu2-I1	2.5757(17)
Cu2-I2	2.5057(17)	Cu2-I1A	2.5784(16)
Cu2-Cu2A	2.561(3)	I1-Cu2A	2.5785(16)
S1-Cu1-S2	115.05(10)	S1-Cu1-O1	122.06(17)
S1-Cu1-O2	83.97(17)	S1-Cu1-N1	125.1(2)
S2-Cu1-O1	84.80(16)	S2-Cu1-O2	112.61(18)
S2-Cu1-N1	119.6(2)	O1-Cu1-O2	140.2(2)
O1-Cu1-N1	69.6(3)	O2-Cu1-N1	70.8(3)
I1-Cu2-I2	118.37(6)	I1-Cu2-Cu2A	60.26(6)
I2-Cu2-Cu2A	176.68(10)	I2-Cu2-I1A	121.12(7)
I1-Cu2-I1A	120.42(6)	Cu2A-Cu2-I1A	60.15(6)

[a]symmetry operation: A) -x+2,-y+2,-z+1.

Table S2 Selected bond distances (Å) and bond angles (°) for **2**[a]

Cd1-I1	2.7422(7)	Cd1-S1	2.7404(18)
Cd1-S2	2.6557(19)	Cd1-O1	2.895(5)
Cd1-O2	2.447(5)	Cd1-N1	2.321(6)
Cd2-I2	2.8882(8)	Cd2-I3	2.7046(7)
Cd2-I4	2.7033(8)	Cd2-I2A	2.8599(7)
Cd2A-I2	2.8598(7)	Cd1···I1B	3.5768(8)
I1-Cd1- N1	115.57(15)	I1-Cd1-O1	78.81(10)
S1-Cd1-S2	97.79(5)	S1-Cd1-O1	161.71(12)
S1-Cd1-O2	76.35(12)	S2-Cd1-O1	74.92(11)
S2-Cd1-O2	83.53(13)	S1-Cd1-N1	135.71(15)
S2-Cd1-N1	103.20(15)	O1-Cd1-O2	118.55(16)
O1-Cd1-N1	62.56(18)	O2-Cd1-N1	67.96(19)

[a]symmetry operation: A) -x+1,y,-z+3/2; B) 1-x,1-y,1-z

Table S3 Selected bond distances (Å) and bond angles (°) for **3**[a]

Cd1-I1	2.7644(8)	Cd1-S1	2.7009(10)
Cd1-S2	2.6694(10)	Cd1-O1	2.751(2)
Cd1-O2	2.564(2)	Cd1-N1	2.283(3)
Cd1···I1A	3.3827(8)		
I1-Cd1- S1	88.23(2)	I1-Cd1 - S2	104.82(2)
I1-Cd1-O1	82.71(5)	I1-Cd1- O2	155.05(6)
I1-Cd1 - N1	122.73(7)	S1-Cd1-S2	89.05(3)
S1-Cd1-O1	159.11(5)	S1-Cd1-O2	71.80(5)
S1-Cd1-N1	136.33(7)	S2-Cd1-O1	75.25(5)
S2-Cd1-O2	90.03(6)	S2-Cd1-N1	108.64(7)
O1-Cd1- O2	120.96(7)	O1-Cd1-N1	63.34(8)
O2-Cd1- N1	68.70(8)		

[a]symmetry operation: A) -x+1,-y+1,-z+2

Table S4 Selected bond distances (Å) and bond angles (°) for **4**[a]

Cd1-I1	2.758(7)	Cd1-S1	2.662(15)
Cd1-S2	2.766(15)	Cd1-N1	2.29(3)
Cd2-I2	2.772(6)	Cd2-S3	2.651(15)
Cd2-S4	2.689(12)	Cd2-N2	2.29(3)
Cd2-O3	2.56(4)	Cd1···I1A	3.415(7)
Cd2···I2B	3.690(7)		
I1- Cd1-S1	110.9(4)	I1-Cd1-S2	91.2(4)
I1- Cd1- N1	117.8(10)	S1-Cd1-N1	100.7(10)
S1-Cd1-S2	96.9(4)	S2-Cd1-N1	137.0(10)
I2-Cd2-O3	160.2(9)	I2-Cd2-S3	111.6(4)
I2-Cd2-S4	86.8(3)	I2-Cd2-N2	119.5(9)
S3-Cd2-S4	102.0(4)	S3-Cd2-N2	101.4(8)
S3-Cd2-O3	83.6(10)	S4-Cd2-N2	134.2(8)
S4-Cd2-O3	77.4(9)	N2-Cd2-O3	66.8(12)

[a]symmetry operation: A) -x+2,-y+1,-z; B) 1-x, 1-y, 1-z

Table S5 Selected bond distances (Å) and bond angles (°) for **5**[a]

Cd1-I1	2.8751(2)	Cd1-I1A	2.8603(2)
Cd1-I2	2.7314(2)	Cd1-I3	2.7150(2)
Cd1A-I1	2.8604(2)	Cu1-S1	2.2600(6)
Cu1-S2	2.2662(6)	Cu1-O1	2.6900(14)
Cu1-O2	2.6081(16)	Cu1-N1	2.0207(17)
Cd1-I1-Cd1A	87.178(6)	I1-Cd1-I2	103.343(7)
I1-Cd1-I3	114.680(8)	I1-Cd1-I1A	92.822(6)
I2-Cd1-I3	116.441(8)	I2-Cd1-I1A	117.538(8)
I3-Cd1-I1A	109.558(7)	S1-Cu1-S2	116.53(2)
S1-Cu1-O1	117.67(3)	S1-Cu1-O2	84.46(4)
S1-Cu1-N1	123.10(5)	S2-Cu1-O1	84.46(3)
S2-Cu1-O2	115.95(4)	S2-Cu1-N1	120.30(5)
O1-Cu1-O2	140.59(5)	O1Cu1-N1	71.52(6)
O2-Cu1-N1	69.08(6)		

[a]symmetry operation: A) -x+1,-y+1,-z+1.
